# Optimum stratum boundaries and sample sizes for Covid-19 data in Egypt

**DOI:** 10.1371/journal.pone.0271220

**Published:** 2022-07-28

**Authors:** Fatma S. Abo_El.Hassan, Ramadan Hamed, Elham A. Ismail, Safia M. Ezzat

**Affiliations:** 1 Statistics Dept, Faculty of Commerce, Al-Azhar University, Girls’ Branch, Cairo, Egypt; 2 Statistics Dept, Faculty of Economics and Political Science, Cairo University, Giza, Egypt; 3 Social Research Center, American University, New Cairo, Egypt; Flinders University, AUSTRALIA

## Abstract

Stratified random sampling is an effective sampling technique for estimating the population characteristics. The determination of strata boundaries and the allocation of sample size to the strata are two of the most critical factors in maximizing the precision of the estimates. Most surveys are conducted in an environment of severe budget constraints and a specific time is required to finish the survey. So cost and time are two important objectives that are taken under consideration in most surveys. The study suggested Mathematical goal programming model for determining optimum stratum boundaries for an exponential study variable under multiple objectives model when cost and time are under consideration. Compared to other techniques, Goal programming has many advantages in resources planning. Determining the required resources to satisfy the desired goals and the effectiveness of the available resources as well as providing best solutions under different amounts of resources are examples of the advantages of Goal programming. In addition the paper used data on Covid-19 to evaluate the performance of the suggested model for the exponential distribution. The study divided the number of new cases diseases into small, medium and high numbers. It also compared the results with the findings in the reports of the World Health Organization. The suggested mathematical goal programming revealed that Egypt was exposed to three waves of infection during the interval (5/3/2020 to 12/8/2021). These results are identical to the actual reality of covid-19 waves in Egypt.

## 1 Introduction

The basic feature of stratified random sampling is that the internally strata units are homogenous, that is, stratum variances should be as small as possible. The equations for determining the optimum stratum boundaries were first provided by Dalenius [1]. Khan et. al. [2] studied the optimum strata width as a Mathematical Programming Problem that was solved using the dynamic programming technique. Khan et. al. [3] studied the problem of optimum stratification and formulated as a MPP assuming exponential frequency distribution of the main study variable. The stratum boundaries are optimum in the sense that they minimize the sampling variance of the stratified sample mean under Neyman allocation. The formulated MPP found to be separable with respect to the decision variables and is treated as multistage decision problem. A solution procedure has also been developed using dynamic programming. Khan et. al. [4] discussed the problem of determining OSB for the study variables with Triangular and Standard Normal distributions. Khan et. al. [5] proposed the method of choosing the best boundaries when strata are formed based on a single auxiliary variable with a varying measurement cost per unit strata by assuming a suitable distribution of the auxiliary variable.

The study is concerned with variables which followed triangular, uniform, exponential, normal, right triangular, Cauchy and power distribution. When the study variable has a pareto frequency distribution, Rao et. al. [6] suggested a procedure for determining optimum stratum boundary and optimum strata size of each stratum. Fonolahi and Khan [7] presented a solution to evaluate the optimum strata boundaries When the measurement unit cost varies throughout the strata and when the variable is exponentially distributed. Reddy et. al. [8] solved the same problem when multiple survey variables are under consideration. Danish et. al. [9] presented optimum strata boundaries as a non-linear programming problem when the cost per unit varies throughout the strata. Reddy et. al. [10] formulated the stated problem under Neyman allocation where the auxiliary variables follow Weibull distributions. Danish and Rizvi [11] suggested a non-linear programming model to determine optimum strata boundaries for two auxiliary variables. Reddy and Khan [12] implemented the problem of optimum stratum boundary for various distributions using R package. The aim of this study is to determine optimum stratum boundary (OSB) using Goal programming approach. Compared to other techniques, Goal programming has many advantages in resources planning. Determining the required resources to satisfy the desired goals and the effectiveness of the available resources as well as providing best solutions under different amounts of resources are examples of the advantages of Goal programming. In addition the paper used data on Covid-19 to evaluate the performance of the suggested model for the exponential distribution. The study divided the new cases disease into small, medium and high numbers. It also compared the results with the findings in the reports of the World Health Organization in terms of times of peak disease. This study aimed at determining the optimum sample size, if necessary, within a certain cost and time.

## 2 Optimum stratum boundaries model

Let the study variable *u* from population stratified into *J* strata and $\bar{U}$ is the estimate of population mean. Let *u*_0_ and *u*_*J*_ be the smallest and largest values of the stratification variable *u*, respectively. The variance of stratified sample mean equal $\sum_{h=1}^{J}W_{h}{\bar{u}}_{h}$ under Proportional allocation,

$$\begin{array}{c} V{\left({\bar{u}}_{st}\right)}_{pro}=\frac{1}{n}\sum_{h=1}^{J}W_{h}S_{h}^{2}-\frac{1}{N}\sum_{h=1}^{J}W_{h}S_{h}^{2} \end{array}$$ (1)

is made as small as possible, in order to determine the intermediate stratum boundaries from the smallest to the largest then ${\bar{u}}_{h}$ is the sample mean in in stratum *h*, *h* = 1, 2, …, *J*, *W*_*h*_ is the proportion of population units in stratum *h* and $S_{h}^{2}$ is the variance of stratum for variable *u* in the *h*_*th*_ stratum. and *n*is the sample size chosen from population *N* and it is equal to $\frac{n_{h}}{W_{h}}$ under proportion allocation. The minimization of variance given by (1) can be expressed as the minimization of

$$\begin{array}{c} V{\left({\bar{u}}_{st}\right)}_{pro}=\sum_{h=1}^{J}\frac{W_{h}^{2}S_{h}^{2}}{n_{h}}-\frac{1}{N}\sum_{h=1}^{J}W_{h}S_{h}^{2} \end{array}$$ (2)

The problem of determining OSB is to find *J*-1 intermediate points in the interval |*u*_0_, *u*_*J*_|, let the distance between the smallest and largest values of the stratification variable *u* is set to be equal

$$\begin{array}{c} u_{J}-u_{0}=q \end{array}$$ (3)

If the study variable *u* has a defined frequency function *f*(*u*), and the boundaries of the *h*_*th*_ stratum are *u*_*h*−1_, *u*_*h*_, then

$$\begin{array}{c} W_{h}=\int_{u_{h-1}}^{u_{h}}f\left(u\right)du \end{array}$$ (4)

$$\begin{array}{c} S_{h}^{2}=\frac{1}{W_{h}}\int_{u_{h-1}}^{u_{h}}u^{2}f\left(u\right)du-\mu_{h}^{2} \end{array}$$ (5)

Where,

$$\begin{array}{c} \mu_{h}=\frac{1}{W_{h}}\int_{u_{h-1}}^{u_{h}}uf\left(u\right)du. \end{array}$$ (6)

using Eqs (4), (5) and (6), *W*_*h*_*S*_*h*_ in Eq (2) can be represented as a function of *u*_*h*_ and *u*_*h*−1_. i.e *f*_*h*_(*u*_*h*_, *u*_*h*−1_) = *W*_*h*_*S*_*h*_. Hence, the objective function is to obtain *u*_1_ ≤ *u*_2_ ≤ … ≤ *u*_*J*−2_ ≤ *u*_*J*−1_. That is adequate to the following MPP:

$Minimize\sum_{h=1}^{J}f_{h}\left(u_{h},u_{h-1}\right)$

*suubject to* *u*_0_ ≤ *u*_1_ ≤ *u*_2_ ≤ … ≤ *u*_*J*−2_ ≤ *u*_*J*−1_ ≤ *u*_*J*_.

Let *q*_*h*_ = *u*_*h*_ − *u*_*h*−1_ ≥ 0 denotes the width of the *h*^*th*^(*h* = 1, 2, …., *J*) stratum. Accordingly, definition (3) is expressed as follows:

$\sum_{h=1}^{J}q_{h}=\sum_{h=1}^{J}\left(u_{h}-u_{h-1}\right)=u_{J}-u_{0}=q$

For *k*^*th*^ point

$$\begin{array}{c} u_{k}=u_{0}+q_{1}+q_{2}+\ldots .+q_{k}=u_{k-1}+q_{k} \end{array}$$ (7)

As a result, determining OSB is the same as determining OSW (optimal stratum width) as MPP:

$$Minimize\sum_{h=1}^{J}f_{h}\left(q_{h},u_{h-1}\right)$$

$$subject\;to\sum_{h=1}^{J}q_{h}=q$$

$$\begin{array}{c} And\;q_{h}\geq 0,h=1,2,\ldots .,J \end{array}$$ (8)

When *h* = 1 the function *f*_1_(*q*_1_) transforms into a function in *q*_1_ only where *u*_0_ is known. in addition, when *h* = 2 the function *f*_2_(*q*_*2*_, *u*_1_) = *f*_2_(*q*_2_, *u*_0_ + *q*_1_) transforms into a function in *q*_2_ only where *u*_1_ is known. As a result, the MPP can be written as a function in *q*_*h*_ as follows:

$$Minimize\sum_{h=1}^{J}f_{h}\left(q_{h}\right)$$

$$subject\;to\sum_{h=1}^{J}q_{h}=q$$

$$\begin{array}{c} And\;q_{h}\geq 0,h=1,2,\ldots .,J \end{array}$$ (9)

## 3 The proposed mathematical goal programming model

The proposed mathematical goal programming model for evaluating OSB and optimum sample size allocation to the strata will be presented in this section. The suggested mathematical goal programming constraints are as follows:

1. The aggregate of the optimum stratum width be equal to the distribution’s range.
2. The cost (not exceeding a fixed limit) was added to the model as objective constrain that will be minimized.
3. Time constraint is taken into consideration as it is needed for the sampling process within a specific range.

To optimally determine stratum boundary, we will allocate the sample to the different strata for variable *u* defined in [a,b]. The problem is to partition *u* into *J* strata such that *a* = *u*_0_ ≤ *u*_1_ ≤ *u*_2_ ≤ … ≤ *u*_*J*−2_ ≤ *u*_*J*−1_ ≤ *u*_*J*_ = *b*, let *u*_*J*_ − *u*_0_ = *q* and define *q*_*h*_ = *u*_*h*_ − *u*_*h*−1_ thus the required stratification points are given as follows:

*u*_*h*_ = *u*_*h*−1_ + *q*_*h*_.

The suggested Goal Programming (GP) approach can be formulated as follows:

find *q*_*h*_, *u*_*h*_, *n*_*h*_, *c*_*h*_ and *t*_*h*_ which:

$$\begin{array}{c} Minimize\sum_{i=1}^{k}\left(dp_{i}+dn_{i}\right),i=1,2,3 \end{array}$$ (10)

$$\begin{array}{c} subject\;to\sum_{h=1}^{J}\frac{W_{h}^{2}S_{h}^{2}}{n_{h}}+dn_{1}-dp_{1}=v \end{array}$$ (11)

$$\begin{array}{c} \sum_{h=1}^{J}c_{h}n_{h}+dn_{2}-dp_{2}=C \end{array}$$ (12)

$$\begin{array}{c} \sum_{h=1}^{J}t_{h}n_{h}+dn_{3}-dp_{3}=T \end{array}$$ (13)

$$\begin{array}{c} \sum_{h=1}^{J}q_{h}=q \end{array}$$ (14)

$$\begin{array}{c} u_{h}=u_{h-1}+q_{h}. \end{array}$$ (15)

$$\begin{array}{c} \sum_{h=1}^{J}n_{h}=n, \end{array}$$ (16)

*h* = 1, 2, …., *J*, *q*_*h*_ ≥ 0 1 ≤ *n*_*h*_ ≤ *N*_*h*_, *dp*_*i*_, *dn*_*i*_ ≥ 0

Where, *k* denoted the total number of goal functions,

*n*_*h*_: Sample size of the *h*^*th*^ stratum

$n=\sum_{h=1}^{J}n_{h}$ :Total sample size

*c*_*h*_: per unit cost of the *h*^*th*^ stratum

*C*: total cost

*t*_*h*_: time per unit of the *h*^*th*^ stratum

*T*: total time

*v*: prefixed variance of the estimator of the population mean

*dp*_*i*_, *dn*_*i*_: positive and negative deviation variables of the *i*^*th*^ goal,

(*i* = 1, 2, 3) is goal functin index where the first goal is to minimize $V({\bar{u}}_{st})$, the second and the third goals are to minimize cost and time of collecting data per unit in each stratum, respectively. $\sum_{h=1}^{J}\frac{W_{h}^{2}S_{h}^{2}}{n_{h}}=V\left({\bar{u}}_{st}\right)$ is assumed if the finite population correction is ignored and *n*_*h*_ denotes thestratum size of the *h*_*th*_ stratum.

## 4 Covid-19 data application

Currently, the entire world is dealing with the world’s most serious health problem, covid-19. The study used covid-19 data which designated by the World Health Organization (WHO) for Egypt. The study used the original covid-19 data from WHO to evaluate the suggested Mathematical goal programming model. The application to covid-19 data adopted the following steps:-

1. The study variable which used is new cases of covid-19 data from the period 5/3/2020 to 12/8/2021 in Egypt. A statistical fit test was applied for the chosen study variable and it was found that The study variable followed an exponential distribution
2. Let the variable under study *u* follows an exponential distribution with parameter *θ* > 0, that is
  $$\begin{array}{c} f\left(u\right)=\{\begin{array}{cc} \theta e^{-\theta u} & ,u\geq 0\\ 0 & otherwise \end{array}. \end{array}$$ (17)
  By using Eqs (4), (5), (6) and (17) the term *w*_*h*_ and $\sigma_{h}^{2}$ can be expressed as follows
  $$\begin{array}{c} w_{h}=e^{-\theta u-1}\left(1-e^{-\theta q_{h}}\right) \end{array}$$ (18)
  And
  $$\begin{array}{c} S_{h}^{2}=\frac{{\left(1-e^{-\theta q_{h}}\right)}^{2}-(\theta q_{h}{)}^{2}e^{-\theta q_{h}}}{\theta^{2}{\left(1-e^{-\theta q_{h}}\right)}^{2}} \end{array}$$ (19)
3. The study suggested mathematical programming in order to calculate the variance when *v* = .965 and take *v* = .965 as an initial value when number of strata *J* = 3 to determine the OSB and optimum allocation into sample strata.
4. As stated before that new cases variable distributed as exponential distribution with *θ* = 0018, *u*_0_ = 0, *u*_*J*_ = 1774 and *q* = 1774 Where *θ* is the parameter for exponential distribution, (*u*_0_, *u*_*J*_) are the observation of smallest and largest values of stratification variable *u* and *q* is the different between largest and smallest value. The study used sample size *n* = 50 (needing to allocate into different strata to estimate average of new cases which presents variable under consideration) from total *N* = 517 which means recorded days from the period 5/3/2020 to 12/8/2021.
5. The suggested Mathematical goal programming is applied when number of strata *J* = 3 representing the approach of classifying the number of new cases diseases into small, medium and high numbers.
6. Cost and time are important objectives of most surveys so the study chosen arbitrary specific range of time, which is equal to 150 hours and the fixed value of cost, which is equal to equal 12000. This is done in order to evaluate the suggested mathematical goal programming when multi-objectives is determined.

Using Eqs (18) and (19), the suggested goal programming model (10–16) when the study variable u is given by Eq (17), can be formulated as follows: Minimize

$$\begin{array}{c} \sum_{i=1}^{k}\left(dp_{i}+dn_{i}\right),i=1,2,3 \end{array}$$ (20)

subject to

$$\begin{array}{c} \left\{\sum_{h=1}^{J}\frac{1}{n_{h}}\left\{e^{-\theta u-1}\left(1-e^{-\theta q_{h}}\right)\frac{{\left(1-e^{-\theta q_{h}}\right)}^{2}-(\theta q_{h}{)}^{2}e^{-\theta q_{h}}}{\theta^{2}{\left(1-e^{-\theta q_{h}}\right)}^{2}}\right\}\right\}+dn_{1}-dp_{1}=v \end{array}$$ (21)

$$\begin{array}{c} \sum_{h=1}^{J}c_{h}n_{h}+dn_{2}-dp_{2}=C \end{array}$$ (22)

$$\begin{array}{c} \sum_{h=1}^{J}t_{h}n_{h}+dn_{3}-dp_{3}=T \end{array}$$ (23)

$$\begin{array}{c} \sum_{h=1}^{J}q_{h}=q \end{array}$$ (24)

$$\begin{array}{c} u_{h}=u_{h-1}+q_{h}. \end{array}$$ (25)

$$\begin{array}{c} \sum_{h=1}^{J}n_{h}=n, \end{array}$$ (26)

*h* = 1, 2, …., *J*, *q*_*h*_ ≥ 0 1 ≤ *n*_*h*_ ≤ *N*_*h*_, *dp*_*i*_, *dn*_*i*_ ≥ 0

## 5 Results and discussion

The study used number of new cases disease for COVID 19 data from Egypt to evaluate the performance of the suggested mathematical programming. Hence, the study solved the suggested goal programming model (20–26) by using a GAMS program and the results are appeared in the following table.

Table 1 summarizes the main findings when suggested goal programming model applied in case of an exponentially distributed random variable and taking into consideration pervious conditions. The suggested model calculated the optimum stratum boundaries for new cases disease divided into three stratum. The first strata was from 0 to 341 cases which presented small number of new cases, the second strata was from 342 to 837 which presented medium number of new cases and the third strata was from 838 to 1774 which presented high number of new cases. The new minimum value of variance.777 which is less than the initial value (0.965) which chosen before. Sample size is divided according to the number of strata to 16, 17 and 17 to first, second and third respectively. As well as dividing the time and cost on the three strata as shown in the above table. The study study elucidated the findings resulted from the suggested mathematical programming using WHO data in Table 2.

**Table 1:** Results for OSB, optimum sample size of the variance function for exponential distribution when *J* = 3.

No. of strata (*J*)	Optimum strata width OSW (*q*_*h*_)	Optimum strata boundary OSW (*u*_*h*_)	Sample size (*n*_*h*_)	(*C*_*h*_)	(*T*_*h*_)	Optimum value of variance
3	341.1	341	16.55 ≈ 16	3808	46	.777
	496.3	837	16.60 ≈ 17	4063.1	49	
	936.6		16.85 ≈ 17	4114	51	

**Table 2:** Strata 1(small cases) strata 2 (medium cases) strata 3 (high cases) and the dates corresponding to three stratum.

	Wave 1	Wave 2	Wave 3
Strata 1	From 15/03/2020 To 03/05/2020	From 01/08/2020 To 18/11/2020	-
Strata 2	From 04/05/2020 To 26/05/2020	From 19/11/2020 To 22/12/2020	From 20/1/2021 To 15/4/2021
Strata 3	From 27/5/2020 To 16/7/2020	From 23/12/2020 To 19/1/2021	From 16/4/2021 To 04/06/2021

The results in Table 2 show that Egypt was exposed to three waves of infection with the emerging corona virus: The first wave started from 15/3/2020 to 3/5/2020, then the number of infected people started to rise from 4/5/2020 to 26/5/2020, and the peak of the first wave was in the period from 27/5/2020 to 16/7/2020, as the number of infected people increased significantly. The second wave started from 1/8/2020 to 18/11/2020, then the number of infected people increased from 19/11/2020 to 22/12/2020, and reached the peak stage in the number of infected people in the period from 23/12 / 2020 to 19/1/2021. As for the third wave concluded from the results of the proposed program, it began with a rise in the number of infected people from 20/1/2021 to 15/4/2021, and those numbers rose and reached their peak in the period from 16/4/2021 to 4/6/2021. The the number of injured decreased in the period from the cut 5 / 6 / 2021 to date of getting data.

**Fig 1:**
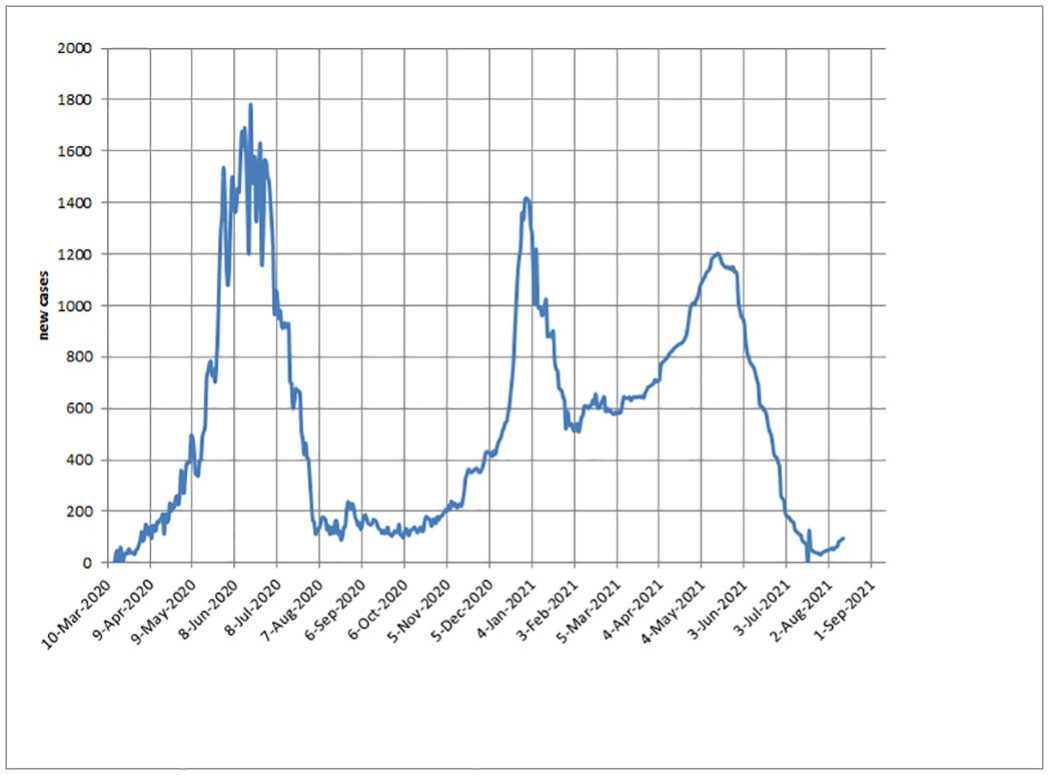
Daily new cases in Egypt reported from WHO data appeared the daily new cases which explain the start date and the end date for the three waves.

The cut points, calculated from the suggested mathematical programming, matching with the waves which appeared in Egypt during the stated period. Fig 1 presented the graph for covid-19 data in Egypt which coincide with the results for the suggested mathematical programming confirming that Egypt faced three waves of covid-19 disease.

New cases reported from WHO data Referring to the web on WHO site, it was found that the Fig 1 corresponds to the results of the proposed program. The suggested mathematical goal programming results are approximately the same compared with the real data which appeared in the WHO real event.

## 6 Conclusion

1. The study suggested mathematical goal programming model for determining optimum stratum boundaries for an exponential study variable under multiple objectives model when cost and time are under consideration.
2. The study collected the data from the world Health Organization reports.
3. Covid-19 data is used to evaluate the performance for the suggested model for the exponential distribution.
4. The study divided the number of new cases into three groups small, medium and high numbers.
5. The study compared the results which calculate from the suggested mathematical programming with the real data from the World Health Organization reports about covid-19 for Egypt.
6. The results show that Egypt was exposed to three waves of infection during the interval (5/3/2020 to 12/8/2021).
7. The results are identical to the actual reality of covid-19 waves in Egypt.
8. Optimum allocation on strata was used as an addition to the suggested program in addition to the cost and time factors.
9. The mathematical goals programming model does not depend primarily on the data, but rather depends mainly on the parameters of the data distribution. Nevertheless, we find that the results are consistent with the reality regarding what Egypt has been exposed to from three waves of the emerging corona virus.
